# A Perspective on Theatre Efficiency in Terms of Theatre Utilisation and Theatre Costs and the Effects of Infection Control Protocols on These During the COVID-19 Pandemic

**DOI:** 10.7759/cureus.31023

**Published:** 2022-11-02

**Authors:** Ghulam Dastagir Faisal Mohammed, Zaina Mansoor, Momin Mohaddis, Prakash Chandran

**Affiliations:** 1 Orthopaedic Surgery, Warrington and Halton NHS Trust, Warrington, GBR; 2 Cardiology, Ysbyty Gwynedd, Bangor, GBR; 3 Orthopaedics, Warrington Hospital, Warrington, GBR; 4 Trauma and Orthopaedics, Warrington and Halton NHS Foundation Trust, Warrington, GBR

**Keywords:** theatre costs, efficiency, utilisation, covid-19, operation theatre

## Abstract

Background and aim

The coronavirus disease 2019 (COVID-19) pandemic has had a significant impact on healthcare systems. Several local infection control methods were put in place, which have now evolved and continued in some form or the other. According to various research, as the time duration for distinct phases in the pathway rose, trauma theatre efficiency reduced. However, there is no literature, to our knowledge, that has explicitly looked at theatre utilisation and cost efficiency compared them and expressed theatre efficiency in these terms. The aim of this article is to study theatre efficiency in terms of utilisation and costs before and during the pandemic and understand the influence of infection control protocols on these.

Materials and methods

The data were collected retrospectively from the ORMIS theatre management software (iPath Softwares, Ohio), from December 2019 (pre-COVID) and December 2020 (COVID). Turnaround time, utilisation time and combined operative time were defined and compared. Costs incurred due to over-running, under-running and turnaround time were compared.

Results

Theatre utilization was 101% during COVID and 86.63% pre-COVID. The average cost of over-running as well as under-running a theatre list during the pandemic was significantly higher.

Conclusion

Optimal theatre utilisation and reduced time between cases improve theatre efficiency. Turnaround time, if reduced, can not only decrease costs but also increase efficiency.Theatre utilisation and efficiency can be maintained even with new infection control protocols, but these are not cost-efficient.

## Introduction

The coronavirus disease 2019 (COVID-19) pandemic has put a strain on healthcare systems. Pressures due to acute trauma decreased as their incidence decreased due to lockdowns, but the incidence of hip trauma was found to increase [1]. Infection control protocols were in place during the pandemic, which have evolved and continued over time. In our hospital, the elective theatres were paused and the staff was relocated to the trauma theatre. All patients were treated as COVID suspects and full infection control protocols were followed, which included full personal protective equipment (PPE) for everyone entering the theatre, a deep cleaning (usage of virucidal fluids to thoroughly clean every surface in the theatre and change of theatre air) after every theatre use, a cooling period of 15 minutes after aerosol-generating procedures (AGP), recovering all patients in the operating room (OR) itself and isolating corridors for the transport of patients. Porters were increased in number so that corridors could be isolated for patient transport. There was a clean runner every theatre session, an extra staff nurse and an extra operating department practitioner (ODP).

Trauma theatre efficiency significantly decreased according to many studies, as the time duration for different events in the patient journey was influenced directly or indirectly by the new infection control protocols that were in place during the pandemic [2-4]. We can quantify the effect of the infection control protocols by comparing several efficiency parameters before and after their introduction. Theatres are the costliest components of secondary care [5] and theatre utilisation is the main managerial measure of performance [6], but cost-effectiveness cannot be completely ignored as a measure of theatre efficiency.

The aim of this paper is to study the trauma theatre efficiency and compare pre-COVID-19 theatre utilisation and costs with that during the COVID-19 pandemic in order to understand the effect of infection control protocols that have influenced these.

## Materials and methods

This is a retrospective quantitative, observational analytic study. There is a single trauma theatre dedicated to orthopaedic trauma at our hospital, which is a busy district general hospital in the United Kingdom. The patient enters the theatre suite complex and then into the allocated theatre, which has an anaesthesia room and an operating room. A responsible person, usually the circulating nurse, using software, records theatre time in a 24-hour format. The following theatre milestones are recorded for every case, namely: Patient sent for, Porter sent for, In-suite, In anaesthesia room (AR), Anaesthetist start time, Anaesthesia ready, In operating room (OR), Procedure start, Procedure finish and out OR.

During non-COVID times, when the theatre staff ring the ward, “Patient sent for” is recorded, closely followed by “Porter sent,” as they immediately inform the porter to fetch the patient. When a patient arrives at the common theatre complex, “In suite” time is recorded. This is then followed by the patient being shifted into the anaesthesia room (AR), and the “In anaesthesia room” time is recorded. A WHO sign-in is done next followed by preparation for anaesthesia. When the anaesthetist begins the process of anaesthesia, “Anaesthetist start time” is recorded, followed by “Anaesthesia ready” when the patient is anaesthetised and ready to be shifted to the Operating room (OR). Once in the operating room, the “In OR” time is recorded, the patient is positioned on the operating table, pre-procedure imaging is done if needed and the surgeons scrub. The “Procedure start” time is recorded when the WHO time out is done. The procedure end time is recorded when the surgical wound is dressed. This is closely followed by the WHO sign-out and shifting of the patient out of the OR. There were several changes in place during December 2020, in view of the pandemic and in accordance with the infection control policies. The most obvious change was that patients used to enter the OR directly through the AR and the WHO sign-in and anaesthesia process was done in the OR itself. The patients were extubated in the OR itself. The pathway remained the same for all patients, regardless of COVID status.

Data were collected from the ORMIS theatre management software (iPath Softwares, Ohio) of all trauma cases operated on in December 2019 (pre-COVID) - Group 1 and December 2020 (during COVID) - Group 2. The software enables the nurses to record theatre timings for every patient. The number of theatre sessions, theatre utilisation, theatre occupied time and theatre turn-around time were compared between the two groups.

Each day was divided into two sessions in both groups. "Theatre utilisation" was defined as the time from the first patient being sent for to the last patient leaving the operating room. "Turnaround time" was defined as the time between the previous patient leaving the OR to the next patient arriving in the anaesthetic room. "Theatre occupied time" was defined as the time from when the patient enters the AR to when the patient leaves the OR.

Under-run lists are those where the theatre list ends with any value of time remaining in the session and over-run lists are where a theatre list over-runs the session time by any value.

The cost of a trauma theatre is estimated to be about £20/minute, which includes £10 for staffing [7] in Group 1, whereas, in Group 2, this was calculated to be £45/minute out of which £15 for staffing, as it was increased and the rest was due to PPE and other requirements. These costs do not include any implant charges.

All data were recorded using Microsoft Excel (Microsoft Corporation, Redmond, WA). Levene’s test was used to test the normality of the data, followed by the independent T-test to assess statistical significance.

## Results

The average time when the first case entered the OR in December 2019 was 10:39:02 and in December 2020, it was 09:36:21 (p = 0.0004). The average time of exit of the last case from the OR in December 2019 was 16:00:50 and in December 2020, it was 17:00:57 (p=0.09). Further results are given in Table 1.

**Table 1:** Comparison of theatre timings between the two groups

Parameter	Group 1 (Pre-COVID)	Group 2 (During COVID)	P-value
Theatre Utilisation	86.63%	101%	0.08
Turnaround Time	13.82%	16.33%	0.41
Theatre Occupied Time	69.59%	79.08%	0.05
Under-Run Lists	67.74%	39.28%	
Mean Under-Run Time	01:54:17	02:28:00	0.28
Over-Run Lists	32.25%	60.71%	
Mean Over-Run Time	01:07:42	01:44:00	0.15

In Group 1, an average of 1.11 cases were attended to per session while in Group 2, this average was found to be 1.03 (p=0.17) The costs incurred due to turnaround time per session in the Pre-COVID group was 580.72 GBP and in the COVID group, it was 1542.85 GBP. The p-value was insignificant at 0.41

According to JJ Pandit et al., efficiency in terms of utilisation = {(fraction of scheduled time utilised)-(fraction of scheduled time over-running)}x(fraction of scheduled operations completed) [8].

The fraction of scheduled time utilised was calculated for each day; this is the theatre utilisation time expressed as a fraction. If all of the theatre time was utilised or the list overran, this fraction is expressed as 1. If there is underutilisation, the number of hours utilised divided by the number of hours available gives us the "fraction of scheduled time utilised".

For the over-utilised lists (where the fraction of scheduled time utilised has been considered as 1), we calculate the number of hours beyond the scheduled time and divide this by the total hours scheduled to give us a "fraction of scheduled time over-running". This is then subtracted from the fraction of scheduled time utilised. For the under-utilised list, the fraction of scheduled time over-running will obviously be zero.

The result is then multiplied by the fraction of scheduled operations completed. This gave us an efficiency ranging from 0 to 1.0. In both groups, we found that all scheduled operations on that particular day were completed in our sample. For Group 1, the average efficiency in terms of theatre utilization using the above formula = 0.70 and for Group 2, the average efficiency in terms of theatre utilisation using the above formula = 0.65. The T-test gave a p-value of 0.51.

According to JJ Pandit et al., Extra cost = Cost of under-running + Cost of overrunning + Cost of cancelled operations [8].

Each day, the cost of over-running or under-running was calculated. The cost of cancelled operations was zero because, in our sample, all scheduled operations were completed in both groups, often at the risk of overrunning a list.

This has been calculated in Table 2 below.

**Table 2:** Calculation of extra costs, costs due to over-running and underrunning of lists

All in GBP	2019	2020	P-value (T-test)
Average cost of over-running per day	2908	9580	0.0004
Average cost of underrunning per day	2639	6619	0.0001
Average extra cost/day according to the formula above	2638/day	8099.50/day	0.000

## Discussion

Theatre efficiency can be expressed in ways more than one, but theatre utilisation seems to be the main managerial measure [6]. The Audit Commission gives theatre utilisation a lot of importance [6] but unfortunately, it is easy to increase this by over-running lists, and this results in cancellations, which in turn are a financial loss. Overrun and underrun lists both decrease theatre efficiency. Under-running causes financial waste and overrunning increases staffing pressures and reduces staff morale [9]. Theatre efficiency can be increased by decreasing the turnaround time, increasing procedure time and achieving optimal theatre utilisation [9]. Due to several new infection control protocols that were affected during the COVID-19 pandemic, theatre efficiency was found to have reduced [2-4], leading to huge financial losses. The infection control protocols like usage of PPE, recovery of patients in the OR and usage of isolated corridors for patient transport directly influenced theatre utilisation and time between cases [2-4]. The infection control protocols also indirectly influenced the theatre's efficiency and costs by increasing the turnaround time and staffing requirements. In our study, the difference in "theatre occupied time" (different from theatre utilisation time) was significant statistically, and this is a reflection of how infection control protocols effects theatre timings, as the recovery of the patient was done in the OR itself during the pandemic, which added to the time a particular patient spent in the OR.

In our study, the difference between mean cases per session remained statistically insignificant between the pre-COVID and COVID groups. This is suggestive of maintained theatre efficiency in terms of the number of cases operated. This was probably due to extra staffing and the ability to over-run a list. We found that our theatre efficiency, in terms of theatre utilisation and turnaround time, was not significantly decreased during the pandemic and in fact, it significantly increased in terms of some parameters like theatre start and end time. This is in contrast to several other studies [2-4].

In a trauma theatre, the utilisation is unpredictable and depends on the previous take. Several factors come into play like availability of beds or patient transport, which have been found to cause the most delay [10] or the availability of staff [11]. Underutilisation is a direct effect of delayed starts, early finishes, and delays in the turnaround of patients [9]. The difference between the two groups in terms of theatre utilisation, although statistically insignificant, was quite large. This could probably be due to the fact that patients were being extubated in the OR itself, and they were shifted out after a cooling period of 15 minutes if at all an AGP was performed. It could also be because we were more efficient, as there was a constant fear of the trauma theatre being overwhelmed due to waiting cases. 

In Group 2, there was a significant early start and delayed end of theatre sessions during December 2020. The early starts were because the morning trauma meeting was very brief in December 2020 due to fewer people in the meeting (restriction of capacity in rooms due to infection control protocols like social distancing) and probably fewer cases. For the same reasons, the ward round was done more quickly. One anaesthetist examined all the patients while the other anaesthetist started the morning huddle, which was not the case during normal times. The last patients exited the theatre significantly later in Group 2 as compared to Group 1. The majority of the lists were over-run during December 2020, giving an impression of theatre utilisation being better. Yet over-run lists decrease theatre efficiency in the long run, leading to staff exhaustion [8].

The turnaround time has been found as a cause of substantial financial waste and most delays were due to delays in sending for the patient [7]. In our study, the difference in turnaround time remained insignificant between the two groups. There is no optimal turnaround time, some studies have accepted a time of 60 minutes [12,13]. Our turnaround time remained well below this threshold in both groups (00:46:30 in Group 1 and 00:46:48 in Group 2). We followed an isolated corridor policy where the path of a patient is to be cleared before transporting the patient to prevent infection. This reduced our delay in sending, as porters were informed earlier and they were ready to transport the patient as a priority. There was a 15-minute warning call given to the ward before the current patient was sent out. The extra ODP was able to do the pre-operative checklist if the patient arrived early before the other patient had left. There was a dedicated cleaning team mobilised soon after closure. These factors were also found to improve theatre turnaround time as per Fletcher et al. [14].

Efficiency was calculated using the formula given by JJ Pandit et al. [8]. We found no statistical difference between the two groups. This could probably be because of the over-utilisation of lists and no cancellations in both groups.

In our study, it was observed that there is a three-fold increment in the cost incurred during the turnaround time per session during the COVID-19 period (1542.8 GBP) as compared to pre-COVID (580.7 GBP). The turnaround time was higher during the COVID-19 period although not statistically significant, which is opposite to what was found in several other studies [2-4]. This could probably be due to the infection control protocols as the theatre had to be deep cleaned after every case. It could also be due to over-utilisation, as staffing levels change significantly post the normal working times and added to this the staffing costs were different during the pre-COVID and COVID times. We believe reducing the turnaround time can significantly reduce theatre costs and improve efficiency.

The calculation of extra costs was done using the formula given by JJ Pandit et al. [8]. It was observed that our over-run lists cost about double that of the under-run list. This is similar to McIntosh et al. [15]. We incurred significantly extra costs during the COVID-19 pandemic, which is due to more over-running lists than under-running lists and over-utilisation. The base cost of running the theatre was higher and the staffing cost per minute was higher, and these will obviously add to the total running costs. Although cancelled operations do cost some amount [8], no operations were cancelled in our samples in both groups in the trauma theatre. This was done at the cost of overrunning a list, which can be seen in both groups. Avoiding overrunning a list, planning a list to avoid cancellations and reducing the turnaround time, in our opinion, will reduce the financial waste in theatre and improve theatre efficiency.

Limitations

This study has several limitations. For example, we have not assessed the delays against patient factors like the American Society of Anesthesiologists (ASA) grade or doctor factors like the grade of surgeon/anaesthetist. A cut-off was not established for turnaround time to calculate the actual loss, the sample size is small and logistic factors like bed availability etc. were not considered.

## Conclusions

Theatre utilisation was high during the COVID pandemic compared to pre-COVID times, which might suggest high theatre efficiency. There was a significant decrease in cost efficiency during the COVID pandemic owing mainly to over-utilisation and high turnaround times, both of which were affected by infection control protocols. The high costs incurred directly or indirectly due to COVID-19 infection control protocols can be mitigated by proper theatre planning and implementation. The infection control protocols have certainly influenced theatre efficiency and if efficiency is to be maintained along with these protocols, it results in significant costs. The managerial measure of efficiency cannot just rely on theatre utilisation, there needs to be more research to bring out a measure of efficiency that incorporates the utilisation, costs and several other complex factors involved in a patient's theatre journey.
